# Independent Use of a Home-Based Telemonitoring App by Older Patients With Multimorbidity and Mild Cognitive Impairment: Qualitative Study

**DOI:** 10.2196/27156

**Published:** 2021-07-12

**Authors:** Madlen Scheibe, Caroline Lang, Diana Druschke, Katrin Arnold, Edwin Luntz, Jochen Schmitt, Vjera Holthoff-Detto

**Affiliations:** 1 Center for Evidence-Based Healthcare University Hospital Carl Gustav Carus and Carl Gustav Carus Faculty of Medicine Technische Universität Dresden Dresden Germany; 2 Department of Psychiatry, Psychotherapy and Psychosomatics Alexianer Hospital Hedwigshöhe St Hedwig Hospital Berlin Berlin Germany; 3 Carl Gustav Carus Faculty of Medicine Technische Universität Dresden Dresden Germany

**Keywords:** telemedicine, aged, multimorbidity, dementia, patient acceptance of health care, health care quality, access, and evaluation, qualitative research

## Abstract

**Background:**

The management of multimorbidity is complex and patients have a high burden of disease. When symptoms of dementia also appear, it becomes even more difficult for patients to cope with their everyday lives and manage their diseases. Home-based telemonitoring may support older patients with multimorbidity and mild cognitive impairment (MCI) in their regular monitoring and self-management. However, to date, there has been no investigation into whether patients with MCI are able to operate a telemonitoring app independently to manage their own diseases. This question has become even more important during the current COVID-19 pandemic to maintain high-quality medical care for this patient group.

**Objective:**

We examined the following research questions: (1) How do patients with MCI assess the usability of the telemonitoring app? (2) How do patients with MCI assess the range of functions offered by the telemonitoring app? (3) Was there an additional benefit for the patients with MCI in using the telemonitoring app? (4) Were patients with MCI able to use the telemonitoring app independently and without restrictions? (5) To what extent does previous experience with smartphones, tablets, or computers influence the perceived ease of use of the telemonitoring app?

**Methods:**

We performed a formative evaluation of a telemonitoring app. Therefore, we carried out a qualitative study and conducted guided interviews. All interviews were audio-recorded, transcribed verbatim, and analyzed using the Mayring method of structured content analysis.

**Results:**

Twelve patients (8 women, 4 men) were interviewed; they had an average age of 78.7 years (SD 5.6) and an average Mini-Mental State Examination score of 24.5 (SD 1.6). The interviews lasted between 17 and 75 minutes (mean 41.8 minutes, SD 19.4). Nine patients reported that the telemonitoring app was easy to use. All respondents assessed the range of functions as good or adequate. Desired functionalities mainly included more innovative and varied educational material, better fit of the telemonitoring app for specific needs of patients with MCI, and a more individually tailored content. Ten of the 12 patients stated that the telemonitoring app had an additional benefit for them. Most frequently reported benefits included increased feeling of security, appreciation of regular monitoring of vital parameters, and increased independence due to telemonitoring. Eight patients were able to operate the app independently. Participants found the app easy to use regardless of whether they had prior experience with smartphones, tablets, or computers.

**Conclusions:**

The majority of examined patients with MCI were capable of operating the telemonitoring app independently. Crucial components in attaining independent use were comprehensive personal support from the start of use and appropriate design features. This study provides initial evidence that patients with MCI could increasingly be considered as a relevant user group of telemonitoring apps.

## Introduction

### Background

Multimorbidity, defined as the simultaneous occurrence of at least two chronic diseases, is a characteristic of the health situation of older people and is common among those in high-income countries [1]. In 2019, more than 58% of adults aged 65 years or over were suffering from multimorbidity in Organisation for Economic Cooperation and Development (OECD) countries and this figure reached up to 70% or more in Germany [2]. The management of multimorbidity is often complex, and patients face several challenges in terms of understanding and self-managing the conditions and medication, regularly monitoring several clinically relevant vital parameters, and coordinating multiple medical services [3,4]. Multimorbidity is also associated with polypharmacy, including the risk of adverse drug events, a decline in physical functioning, or increased health care utilization such as emergency admissions [5-9]. This often results in decreased quality of life, including psychological distress [1,5,9]. Moreover, mental illnesses such as anxiety and depression are more common in patients with multimorbidity [3]. When symptoms of dementia also appear, it becomes even more difficult for patients to cope with their usual and independent tasks in daily life and manage their own diseases [3]. Additionally, symptoms of dementia act as risk multipliers across all age and morbidity strata, leading to worse health outcomes [10]. It is estimated that in 2019, nearly 20 million people had dementia in OECD countries. This number will more than double by 2050 if current developments continue [2]. Mild cognitive impairment (MCI) represents a preclinical, transitional stage between healthy aging and dementia. MCI has been shown to affect 10%-15% of the population 65 years and older. Each year, 10%-15% of people with amnestic MCI progress to Alzheimer disease compared to only 1%–2% of the healthy older generation [11].

Telemonitoring can play an important role in coping, compensating, and supporting cognition [12,13]. Regular home-based telemonitoring may support older patients with multimorbidity and MCI in their self-management and regular home monitoring of clinically relevant vital parameters. Furthermore, telemonitoring may help patients to feel more secure, remain longer and independently in their familiar home environment, and increase overall quality of life. At the same time, telemonitoring helps to relieve the burden on formal and informal caregivers [12-16].

There are already a large number of telemonitoring apps available for different kinds of chronic diseases, with corresponding usability and acceptance evaluations that have been summarized in several systematic reviews [17-20]. However, the number of existing studies and evaluations on telemonitoring apps focusing on multimorbidity is currently limited [21-23]. Another issue that has not yet been investigated is whether patients with MCI are able to use a telemonitoring app independently, and how they assess usability and acceptance. This target group is considerably large and so too is the associated potential for improved care. Therefore, the aim of this study was to help close this research gap.

Within our study, “usability” was defined as “the extent to which a product can be used by specified users to achieve specified goals with effectiveness, efficiency, and satisfaction in a specified context of use” [24], whereas user acceptance was defined as the “attitude towards a particular situation” [25].

The question of whether patients concurrently suffering from multiple chronic diseases and MCI would be able to use telemonitoring apps has become even more important in recent months due to the COVID-19 pandemic. Telemonitoring apps can be of great value, especially for vulnerable patient groups such as the chronically ill, as patient care was only possible to a limited extent during the lockdown. For example, outpatient visits were cancelled or postponed, and the availability of in-person support services was reduced [26,27]. In addition, especially in such isolation situations, people with chronic illness depend on close health care to prevent serious complications or even death resulting from those complications [28-30]. The health care system needs to respond to the needs of patients suffering from chronic noncommunicable diseases, which are the majority of conditions [31]. Disease-tailored and easy-to-use home-based telemonitoring solutions could be a suitable measure to continue the care for chronically ill patients while maintaining the legally required social distance, and to give them the secure feeling of being well cared for [28,29,32].

### Study Aims and Research Questions

This study was part of the feasibility study “Autonomy despite multimorbidity in Saxony through patient empowerment, holistic care for older people with networking of all regional institutions and service providers” (ATMoSPHAERE) performed between October 2015 and June 2019. The main aim of the study was the iterative development of a technology-based information and communication platform enabling an intersectoral networking of treating physicians in practices, nurses, therapists, social services, and patients with multimorbidity and their caregivers. The comprehensive study design has already been reported elsewhere [33,34]. Within the study, a telemonitoring app for patients was developed (see Description of the Telemonitoring App in the Methods section). The aim of this study was to perform a formative evaluation of the telemonitoring app from the perspective of older patients with multimorbidity suffering from MCI.

In detail, we examined the following research questions by means of a qualitative study: (1) How do patients with MCI assess the usability of the telemonitoring app? (2) How do patients with MCI assess the range of functions offered by the telemonitoring app? (3) Was there an additional benefit for the patients with MCI in using the telemonitoring app? (4) Were patients with MCI able to use the telemonitoring app independently and without restrictions? (5) To what extent does previous experience with smartphones, tablets, or computers influence the perceived ease of use of the telemonitoring app?

Overall, our formative evaluation had two aims. The first aim was to examine the usability of the app and possibilities of independent use to evaluate its perceived ease of use. The second aim was to evaluate the content and the additional benefits resulting from the use of the telemonitoring app to assess its perceived additional benefits. Both evaluation issues were equally relevant for an adequate evaluation to develop an individually tailored telemonitoring app.

## Methods

### Study Design

We opted for a formative evaluation to assess a telemonitoring app that was under development while performing this study. Patient feedback from the interviews provided important aspects for the further iterative development process of the telemonitoring app in line with the needs of the target group [35-37].

When planning the substudy, we had to consider what could be expected of this target group. We had to make sure that the formative evaluation would not lead to excessive demands over and above those caused by actual usage of the telemonitoring app. Keeping this in mind, we decided to apply only one iteration stage and chose the qualitative method of guided interviews for this target group.

We opted to use personal interviews instead of questionnaires since we expected a certain degree of insecurity and restraint toward the research topic among the participants due to a possible lack of previous experience [38]. We used this method as it was particularly advantageous for our vulnerable patient group. This method enabled a personal conversation, thus facilitating a relationship of trust to be established; if necessary, one could explain something again or, in case of ambiguities, one could specifically ask for more information. Additionally, this approach enabled us to adapt the interview guideline flexibly according to the participants and their individual characteristics (eg, health status, individual burden of disease, life situation), and their previous experience in handling tablets, smartphones, or telemedicine solutions [39-41]. At the same time, the use of an interview guideline enables comparisons between the interviews and also prevents storytelling from digressing too far [40,42,43].

### Recruitment of General Practitioners and Study Patients

Potential general practitioners were partners within a network of accredited academic teaching practices. Their practices were located in the city of Dresden, Germany, with approximately 560,000 inhabitants. Interested general practitioners were recruited in network meetings. They were informed about the study and signed a declaration of consent form.

Study patients were recruited by the general practitioners. Study nurses screened patients by applying the validated measuring instruments Mini-Mental State Examination (MMSE; assessment based on [44], German version [45]) and the Clock-Drawing Test [46] to assess cognition, as well as the Timed Up & Go test [47] to assess the mobility of potential study patients. The crucial factor for study inclusion was the degree of cognitive impairment, which was assessed by an MMSE score of 20-26 at the baseline assessment. Patients who were found to be eligible and met the inclusion criteria (Textbox 1) were informed by their general practitioner about the study and received written information. After patients decided to participate, they signed a declaration of consent form. Patients could withdraw their consent at any time.

Study patients were asked about their readiness for a personal interview after using the telemonitoring app for at least 2 months. After the study patients agreed to be interviewed, a researcher informed them about the interview details. Participants were included regardless of whether or not they had prior experience with the use of smartphones, tablets, or computers.

**Study inclusion and exclusion criteria.**

**Inclusion criteria**

- Age ≥65 years
- Multimorbidity (presence of at least two chronic diseases)
- Mild cognitive impairment defined by a Mini-Mental State Examination (MMSE) score between 20 and 26 or mild dementia according to International Classification of Diseases (ICD)-10
- Capable of understanding patient information and consenting to the study
- Independent operation of television via remote control and/or computer/laptop three or more times per week
- Unimpaired hearing
- Sufficient motoric and sensory speech ability
- Sufficient eyesight to follow a television program easily

**Exclusion criteria**

- Missing capacity of consent
- Unable to speak German fluently
- Moderate to severe dementia defined by an MMSE score <20 or according to ICD-10
- Motoric impairment (Timed-Up & Go test ≥30 seconds in initial measurement, 20-29 seconds in two repeated measurements)
- Severe psychiatric comorbidities (eg, schizophrenic psychoses, addictions)
- Currently participating in a comparable telemonitoring program or participation within the last 12 months

### Description of the Telemonitoring App

**Figure 1:**
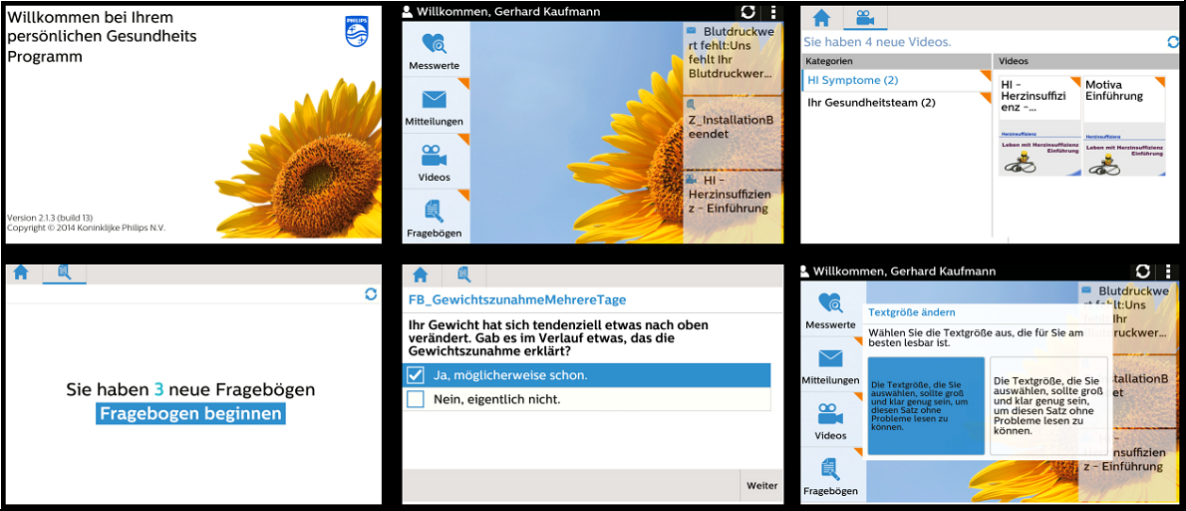
Screenshots of the user interface of the telemonitoring app.

The telemonitoring app was provided by the technical project partner Philips Medical Systems GmbH (hereafter Philips) and consisted of the telemonitoring software Motiva, the telemonitoring hardware in the form of a tablet (ASUS ZenPad 7.0 or Samsung Tab 4), as well as a Bluetooth-enabled sphygmomanometer. Figure 1 shows images of the telemonitoring app’s user interface.

After study inclusion, study patients were instructed at home on the use of the hardware and software by a technician from the German Red Cross. In addition, they received a user manual where essential functions were explained in an easy-to-understand way.

Table 1 provides an overview of the functionalities offered by the telemonitoring app and the corresponding tasks that patients were responsible for performing according to the general practitioner’s treatment regime.

**Figure 2:**
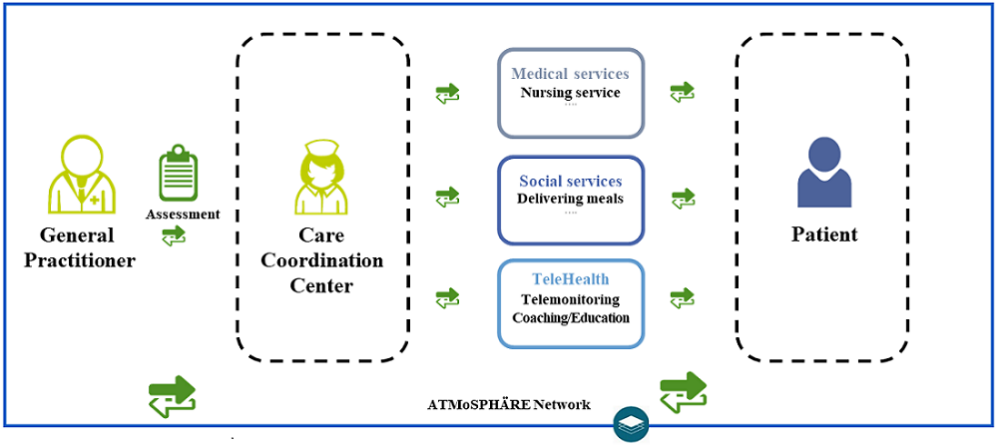
ATMoSPHAERE network including the telemonitoring app. ATMoSPHAERE: Autonomy despite multimorbidity in Saxony through patient empowerment, holistic care for older people with networking of all regional institutions and service providers.

All collected patient data were transferred to the ATMoSPHAERE platform and could be viewed by the responsible general practitioner (Figure 2).

**Table 1:** Overview of functionalities of the telemonitoring app and patient tasks to be performed.

Functionalities offered by the telemonitoring app	Patient tasks to be performed
Measurement of vital data with provided measuring devices at predefined times according to the general practitioner’s treatment regime; data are automatically transmitted to the tablet and to a German Red Cross care coordination center for intervention necessity assessment	Once per week: measurement of blood pressure and heart frequency
Continuous weekday monitoring of measured vital data values by case and care managers at the care coordination center; these managers contact patients in case of exceeding vital data values for possible therapeutic intervention (thresholds predefined by general practitioner)	Completing intervention questionnaires providing information about the reasons for deviation to the case and care managers to derive possible therapeutic interventions
Overview of measured vital data and vital data charts	Not applicable
Regular provision of patient questionnaires	Once at the beginning: completing a questionnaire on general health conditions Once per week: Completing questionnaires on treatment modifications, medication adherence, sleeping habits, pain, alcohol and tobacco consumption, and the disease-specific health status (eg, chronic heart failure and type 2 diabetes) Depending on individual needs: completing additional questionnaires sent by the case and care managers (eg, on the topics of nutrition and depression)
Provision of educational and training material, particularly instructional videos for individual chronic diseases	Not applicable
Receiving messages from the case and care managers (eg, reminder of measurement or answering questionnaires, video recommendations, or congratulations on milestones)	Not applicable

### Theoretical Framework and Interview Guideline

As a basis for the guided interviews, we developed a uniform guideline with open-ended questions (see Multimedia Appendix 1). A specially created set of usability criteria and comprehensive overview of acceptance factors with explicit regard to the requirements of older people concerning telemedicine apps has previously been developed and published by one of the authors of this study [38,48,49], which has been used in several studies to evaluate telemonitoring apps for older people [50-53]. These criteria served as the theoretical framework for guideline development. Guideline development and formulation of interview questions also were established following guidelines from the relevant methods literature [39,54,55].

### Data Collection

The patient interviews were conducted between June 2016 and December 2017. Continuous study inclusion facilitated the interviewing of new study participants throughout the study period. All study patients received the same telemonitoring equipment (tablet, sphygmomanometer). To enable comparisons among patients, all interviews were based on the same interview guideline. All patients opted for a face-to-face-interview at their homes. All interviews were audio-recorded.

### Data Evaluation

All interviews were transcribed verbatim. The transcripts then served as the foundation upon which consecutive data analysis was performed. All interviews were analyzed applying the method of structured content analysis developed by Mayring [56]. This is the central content analysis technique and allows for an association between the deductive and inductive creation of categories [56,57]. The analytical focus was on designing a system of categories and subcategories, as well as their characteristics [56], which in turn served as structural dimensions.

Coding started with the development of an initial deductive category system derived from the questions in the interview guideline, discussed and agreed upon within the research team (DD, KA, MS, EL). Two analyzing researchers (EL, MS) coded two interviews independently to further develop the category system. These researchers differed in age and gender to allow for diversity of perspectives in the context of data analysis. This was then discussed by the entire research team and consolidated. The consolidated category system was then used by two researchers (EL, MS) as the basis for coding all interviews. During analysis, the coders independently specified, modified, or removed categories based on the text material. Missing but relevant categories were added inductively based on the transcripts. This process was continued until saturation of the category system was reached (ie, no new categories emerged) [57].

The inductive development of categories was carried out as follows. Based on the textual material in the transcripts, units of meaning formed the units of analysis. Relevant content of the units of meaning was paraphrased to generate a category label. In accordance with recommendations from the methodological literature, a low level of abstraction was initially selected for the generation of category labels [56]. It was scaled down in the course of analysis and further review of the text material. Subsequently, the abstraction level of the different categories was harmonized to reach a final uniform abstraction level of the category system. Finally, the assignment of individual text sections to the respective categories was reviewed again by the entire research team. Differences in coding were discussed and resolved by consent. If necessary, text segments were recoded accordingly.

Our approach complied with the principles of openness and investigator triangulation within qualitative research [40,54]. For data analysis, the software MAXQDA (MAXQDA Plus 12 portable) was used.

The chosen method allowed us to individually adapt the interview guideline to the actual interview and to the aspects presented as relevant by the participants. In turn, this resulted in interviews where we were not able to ask all possible questions, patients did not answer questions even after the question was repeated, or where the participants themselves added new aspects.

### Ethics Approval

The ethics committee at Technische Universität Dresden (approval number EK 1012016) approved the study.

## Results

### Patient Characteristics and Interview Duration

Of the 19 participants that met the inclusion criteria of our study, 12 agreed to be interviewed. To achieve the greatest heterogeneity possible within our study sample, we interviewed all 12 patients. The interviews lasted between 17 and 75 minutes (mean 41.8 minutes, SD 19.4). Table 2 shows the patient characteristics of the selected cohort.

Ten study patients used an Asus ZenPad 7.0 tablet and two study patients used a Samsung Tab 4.

**Table 2:** Characteristics of the interviewed patients and the average result of the Mini-Mental State Examination (MMSE) (N=12).

Patient characteristics		Sample value
**Gender, n (%)**		
	Male	4 (33)
	Female	8 (67)
**Age category (years), n (%)**		
	65-74	2 (17)
	75-85	9 (75)
	≥86	1 (8)
Age (years), mean (SD)		78.7 (5.6)
**Marital status, n (%)**		
	Single/widowed	6 (50)
	Married/cohabitation	6 (50)
**Number of comorbidities, n (%)**		
	2-8	7 (58)
	≥9	5 (42)
**Comorbidity, n (%)**		
	Essential (primary) hypertension	10 (83)
	Type 2 diabetes mellitus	5 (42)
	Chronic ischemic heart disease	4 (33)
MMSE score, mean (SD)		24.5 (1.6)

### Assessment of Usability by Patients With MCI

#### Usability of the Telemonitoring App

With respect to experience during the initial phase of use, 7 of 10 patients reported that the telemonitoring app was difficult to understand at the beginning. Six of 11 patients reported a feeling of insecurity.

Well, in the beginning I was also doubtful: Can you do it or not or are you doing something wrong? And I was just told, if you entered something wrong, you can always do it again.

Patient 47

Well, I first had to fumble a bit with the thing. I have just a normal phone here. [...] Now it’s fine.

Patient 214

After talking about the initial phase, we asked patients to describe the current usage situation. We observed a clearly positive development. Nine of the 12 patients reported that the telemonitoring app was easy to use at this time: “I find it easy to use. At the beginning [...] I was also anxious.” [Patient 47].

After being asked about difficulties in using the telemonitoring app, one patient answered: “Well, actually nothing more. But the first time, I hadn’t really gotten into it. But that was a long time ago.” [Patient 214].

With respect to individual usability aspects of the telemonitoring app, 7 of 10 patients understood the presentation of the contents of the telemonitoring app well (ie, the used figures and language). Seven of nine patients rated the used symbols as easy to understand.

*The questions which are written in the blue box are clear. And then there’s “Start” or “Back” if I saidsomething wrong.* [Patient 37]

Yes, that's all explained in the manual, yes. It’s a nice red triangle, you practically have to press on it and you just need to read it properly.

Patient 47

The size of images and illustrations (8/8), the font size (12/12), as well as the color contrast (10/10) were rated as perfectly appropriate by all patients who answered these questions within the interviews.

As another aspect of usability, the effort to make inputs was perceived to be less burdensome by almost all patients (10/11). Seven of 11 patients pointed out that this was made possible due to the automated transmission of vital data from the measuring device to the tablet. Six of seven patients rated the menu navigation as simply structured and easily comprehensible.

#### Tablet Usability

When examining the usability of a telemonitoring app, it is also worth considering the ease of use of the corresponding hardware in terms of the tablets.

Nine of 10 patients expressed that the tablet was easy to use. However, the patients reported functional problems with the hardware. The very slow startup of the tablet, including difficulties in finding an internet connection (7/9), and the arbitrary change of the device into flight mode, including difficulties in transferring the measured vital data to the ATMoSPHAERE platform (4/9), were most frequently mentioned. Three of nine patients reported difficulties in operating the On/Off switch and the same number were bothered by a low battery life or long charging time of the tablet. These functional issues had a negative impact on the overall satisfaction with the telemonitoring app provided, with only six of nine participants having reported being very satisfied or rather satisfied.

Interviewer*: So, on the whole, would you say it’s fun to use that or rather not so much fun?*

Patient 225*: If it worked right, I would enjoy it […].*

Interviewer*: Yes, but as it is now?*

Patient 225*: [...] I always approach it with a bit of mixed feelings.*

#### Range of Functions of the Telemonitoring App

Six patients assessed the telemonitoring app’s range of functions and all rated it is as good or adequate. They also named desired functionalities to be included in the app. Three items were most frequently mentioned. The first item involved provision of more innovative and varied educational materials:

Yes, but that's always the same, isn’t it? [...] It is always the same there. The woman, I don’t know her name and blood sugar and stuff. That's something that gets on my nerves

Patient 214

The second item was related to a better fit of the telemonitoring app to the specific needs of patients with MCI:

The only thing I have, that really concerns me is my short-term memory. And that is not being treated here. [...] I wish it were, because many people feel that way. [...] And then also about the operating instructions, you could get something every 2 months or 3 months short, a small article, on one page, that’s enough: “We’ll tell you again about the operating instructions.”

Patient 245

Support for dementia development is not in here, is it? But I still hope that maybe at some point it will be further developed, that maybe some suggestions will be implemented.

Patient 55

The third item was related to having more individually tailored contents of the telemonitoring app in terms of more individualized questionnaires and response categories or by considering additional diseases within the range of functions:

A huge number of food suggestions and so on. But then, maybe other things that would be more interesting. They cannot be queried. I don’t know. You can’t write anything in it by yourself.

Patient 245

### Additional Benefits, Negative Effects, and Changes in Everyday Life of Patients With MCI

#### Additional Benefits of the Telemonitoring App

Ten of the 12 patients stated that the telemonitoring app has an individual additional benefit for them. Eight of the 12 patients stated that they have an increased feeling of security owing to the regular transmission of vital data, and the knowledge that case and care managers are checking their values and will contact them in case of exceeding critical values. Patients reported: “They’ll take care of me” [Patient 225] and “[...] one is monitored and that is not wrong in my opinion” [Patient 179]. The fact that some of the interviewees did not have supporting family members in the direct neighborhood reinforced that feeling.

Five of the 12 patients regularly measured their blood pressure only since having started using the telemonitoring app and appreciated that kind of monitoring: “Well, you either just do it or you forget and here, I do it” [Patient 214].

Four of the 12 patients rated as positive the possibility of being able to monitor blood pressure independently of the general practitioner’s visit according to their individual needs/feelings. According to one patient, this leads to “[…] independence because you know it’s your blood pressure, everything is fine. And you can just go. […] in the beginning someone always had to go shopping with me” [Patient 47].

Furthermore, the following aspects were positively rated by the interviewed patients: the availability of more health-related data as an improved basis for general practitioners’ treatment decisions, an individualized overview of the development of vital parameters, and the perception of the telemonitoring app as a welcome change to everyday life.

#### Negative Effects of Using the Telemonitoring App

Aside from the additional benefit of telemonitoring app use, which was central for the majority of interviewed patients, 2 of the 12 patients also reported negative effects in using the telemonitoring app. The study-related, more frequent measurement of vital data led to uncertainty, because patients could not properly classify fluctuating values due to their lack of expertise. Patients were aware that case and care managers from the German Red Cross intervened in instances of exceeding values, but even slight fluctuations seemed to lead to uncertainty. In addition, differences in the values between the devices used in the study and patients’ own measurement devices were reported to be disturbing.

#### Changes in Everyday Life From Using the Telemonitoring App

We also asked the participants to what extent their everyday life has changed due to the use of the telemonitoring app. Six of nine patients reported that study participation and regular measurements did not represent any significant changes: “[…] you accept this early in the morning, this 5-minute thing in no way makes it difficult” [Patient 179]. Three of nine patients stated that the telemonitoring app even simplified their everyday life. They rated the effort to use it as very little and its integration into everyday life as simple.

### Ability of Patients With MCI to Use the Telemonitoring App Independently

Almost all patients (10/11) had received further support after their initial introduction to the telemonitoring app at home. Most commonly (8/11), they received telephone support from the case and care managers at the German Red Cross or got help directly from Philips, or a German Red Cross technician visited patients at their home in the case of serious problems. Four of 11 patients stated that they had used the user manual, which had been given to them at the beginning of use. Besides the support by project staff, family members assisted patients in using the telemonitoring app. For 3 of 11 patients, the partner/spouse and for 4 of 11 patients, other family members such as children and grandchildren played an important role in handling the app. In some cases, patients were not using the telemonitoring app themselves: Patient 55 (supported by daughter), Patient 61 (supported by wife), as well as Patient 68 and Patient 99 (supported by husbands). Patient 68 and Patient 99 showed the lowest MMSE scores (22) within the study population; Patient 55 showed an MMSE score of 26 and Patient 61 had a score of 25. Patients 61 and 68 each had a supporting spouse; these spouses had been included in the overall study (but not in this substudy) and had each achieved a higher MMSE value themselves. This might be the main reason that these supporting spouses took care of the transmission of vital data and other aspects of study participation.

The other eight patients were able to operate the telemonitoring app independently, in spite of their MCI.

### Influence of Previous Experience With Smartphones, Tablets, or PCs on Perceived Ease of Use of the Telemonitoring App

Three of the 12 patients reported previous experience with a computer but not with a smartphone or a tablet. One patient stated previous experience with a smartphone/tablet but not with a computer. Two patients had already used both a computer and a smartphone/tablet. Two patients had never used a computer, smartphone, or tablet before. Four patients did not comment on this question.

In the context of evaluation, we considered separately to what extent people without prior experience might have had greater difficulties in using the telemonitoring app. The participants found the app easy to use regardless of whether or not they had prior experience, and there were no clear differences in the assessment of the individual usability aspects examined. Only one of the 12 patients, who already had previous experience with a smartphone, found the telemonitoring app difficult to use.

## Discussion

### Main Findings

To our knowledge, our study is the first to investigate whether patients with MCI are able to operate an app for monitoring their multiple chronic diseases, and how they evaluate its usability and additional benefits for their everyday life.

As one main result, we were able to show that the majority of examined patients with MCI were capable of operating a telemonitoring app independently. However, we also found the following framework conditions and features of the telemonitoring app to be crucial preconditions for independent telemonitoring by patients with MCI, resulting in high perceived ease-of-use: personal support and design features.

All patients, with one exception, received further support after their initial introduction to the telemonitoring app at their home. Most commonly, they received telephone support from the case and care managers at the German Red Cross or directly from Philips, or a German Red Cross technician visited the patients at home in the case of major problems. Thus, a personal introduction and the availability of constant and familiar contact persons are important in decreasing the perceived effort of use and increasing acceptance among this target group. Our previous studies with older patients suffering from chronic diseases also found this to be a key acceptance factor [38,49]. These factors have already been assessed as crucial in the “Senior Technology Acceptance & Adoption Model (STAM)” [58] and by Schmid et al [59]. Furthermore, the availability of constant and familiar contact people also reduces the fear that using technology may result in loss of human contact [12].

Perceived ease of use and perceived additional benefit are the main impact factors on user acceptance within various well-known technology acceptance models [58,60,61]. The following design features of the telemonitoring app examined resulted in high usability, and therefore in high perceived ease of use, for patients with MCI: (1) use of understandable semantics (eg, no foreign language words or technical terms that are not generally understandable); (2) use of easily understandable outputs and displays; (3) easily understandable and self-explanatory menu structures; (4) easily understandable navigation to the desired content of the telemonitoring app; (5) sufficient sizes of fonts and illustrations; (6) sufficient color contrast; (7) low input effort through automatic transmission of blood pressure values; and (8) clearly understandable feedback from the platform on (incorrect) input.

The telemonitoring app examined largely met the criteria that earlier studies have shown to be crucial for a high level of usability for older users [12,38,48,49,59]. This in turn led to higher user adherence [62]. However, usability was partly restricted by functional problems of the hardware that resulted in patient dissatisfaction. These reliability problems can result in a lack of trust and less extensive use or even end of use [34,63,64]. Hardware robustness and a stable internet connection are two key requirements for enabling the use of a telemonitoring app. Both have been highlighted as crucial issues in many studies [53,65,66]. If both requirements are not met, independent operation and use of support services are significantly more difficult or even not possible. Vulnerable individuals, especially those with cognitive impairment, could become worried by experienced difficulties. This can lead to the fact that otherwise useful telemonitoring apps may not be beneficial for these patients. Additionally, a fundamentally high usability of the telemonitoring app and permanently available contact persons for technical questions are crucial to relieve the burden on informal caregivers. For older patients with MCI, these people are often the first point of contact for questions and usage problems, and difficulties would thus put them under additional strain [67,68].

As another main result, our study revealed that the participants found the telemonitoring app easy to use regardless of whether or not they had previous experience with the use of smartphones, tablets, or computers; this presupposes that the telemonitoring app has the relevant design features mentioned above and that personal support is continuously available on weekdays.

However, our study results also discovered that some of the patients hardly ever worked with the app themselves, and their relatives predominantly operated the app instead. In future studies, patients experiencing difficulty could possibly operate the telemonitoring app together with their spouses or other relatives living close by. This could contribute to a feeling of security and support for both sides. However, the use of the telemonitoring app can be problematic for patients who live alone and do not receive any support from other individuals. Therefore, future research should examine how a telemonitoring app with personal support should be designed and function to enable independent usage by this vulnerable target group. In this context, future telemonitoring apps should be developed in close cooperation with patients with MCI to consider their needs and perspectives comprehensively. This kind of codesigning has also been emphasized as a central requirement for a high degree of usability and user acceptance within several studies [14,69-72]. In addition, greater patient involvement can lead to empowering effects among this patient group [70,71].

The majority of patients with MCI perceived the telemonitoring of their state of health as beneficial. Most frequently, the patients reported an increased feeling of security. Other studies have also shown this aspect as a significant benefit of telemonitoring app usage by older patients [73,74]. In addition, the possibility of being able to measure blood pressure independently of a general practitioner visit led to more autonomy and independence according to the opinion of four study patients. Several studies have shown autonomy of patients to be a positive outcome of telemedicine interventions, as indicated in a recent review by Kruse et al [75]. This effect is especially valuable for multimorbid, older patients with MCI who are facing several challenges in terms of understanding and self-managing their health conditions [8,9]. The management of multimorbidity is often complex, and patients face several challenges in terms of understanding and self-managing the conditions and medication, regular monitoring of several clinically relevant vital parameters, and coordinating multiple medical services [3,4].

Our study also showed that patients with MCI desired greater consideration of individual characteristics within the telemonitoring app. They explicitly asked for more individualized questionnaires and response categories. They also stated that additional diseases should be considered in the development of the telemonitoring app. Other studies also verified an individually adjustable and modular content of the app to be crucial for increasing perceived usefulness among the highly heterogeneous older population with chronic illnesses [38,48,49,59,69,73,76]. The challenge for the app development process is to enable customization and guarantee high usability at the same time. The consideration of artificial intelligence or self-learning approaches could be useful in this matter.

This study was a formative evaluation as part of an iterative development process. The telemonitoring was further developed in accordance with the feedback from the interviews. In the course of the project, patients were also able to use Fresh Minder apps for memory training [77]. Moreover, additional questionnaires and care plans (eg, for pain, dizziness, sleep, and activity) were developed and implemented, and offers for potentially helpful social and nursing services were added.

With regard to coping with the effects of the COVID-19 pandemic, our results have shown that telemonitoring of older patients with multimorbidity and MCI is feasible. Studies have also shown that the pandemic has changed health care toward increased acceptance and utilization of telemedicine by both patients and providers [29,32]. Furthermore, telemonitoring can help to reduce fears, insecurities, and the feeling of social isolation among those affected [29], which also plays an important role in coping with the COVID-19 pandemic.

### Implications for Future Research

Telemedicine solutions for patients with MCI had already focused on the following topics, which have been systematically reviewed by Lorenz et al [13]: preservation or improvement of memory performance [78,79], app-based memory training [77,80], preservation of fitness and agility [81], preservation of an independent way of life [82], and provision of information about dementia [79]. All of these topics focused exclusively on patients with cognitive impairment. Future research should focus more on influencing factors enabling patients with multimorbidity and MCI to take an active and participatory role within their treatment process by using telemonitoring apps. Furthermore, research is needed to examine up to which severity stage of dementia patients are able to use a telemonitoring app. Therefore, more qualitative and quantitative studies are required to explore this topic in further detail.

Future studies should be performed in a controlled design. It would be expedient to investigate whether and how telemonitoring itself, and the increased feeling of security and independence, affect the number of general practitioner visits, vital parameters, disease progression, medication adherence, emergency admissions or admissions to nursing homes, quality of life, depression, or empowerment. Furthermore, a health economic evaluation would be useful to examine whether the use of such a telemonitoring app can lead to better care at the same costs or with cost savings.

### Implications for Practice

Our study provides initial evidence for the usage of a telemonitoring app by individuals with MCI. We showed that patients with multimorbidity and MCI can be considered as a target group for the use of telemonitoring apps if the above-mentioned conditions are met. For general practitioners and other health professionals, it is important to carefully select which patients are suitable for using such technology and to intensively discuss this option with the patients. Any existing concerns can also be addressed in this context. The patient’s needs and own perceptions, including in the sense of self-selection, are essential requirements for successful use. For individuals with various preexisting chronic conditions, it is essential to carefully determine whether such an app offers effective support. Basically, such apps must fit into the individual treatment pathway and should not represent an additional burden for either the patient or the physician. In this context, it should be taken into account whether relatives are available or whether the patient lives alone and how this might affect use of the telemonitoring app. In addition, the general practitioner or health care professional and the patient should regularly assess and jointly decide whether or not such an app remains suitable.

Overall, the COVID-19 pandemic has caused a rethinking of many issues, which may lead to greater receptiveness among this patient group and treating physicians for such apps.

### Strengths and Limitations

This study was carried out as part of the ATMoSPHAERE project, which ended in June 2019 and focused on older patients with MCI. To our knowledge, our study was the first to investigate whether patients with MCI are able to operate a telemonitoring app for managing symptoms of their multiple chronic diseases. We were able to show that the majority of examined patients with MCI were capable of operating a telemonitoring app independently. With regard to ensuring adequate care for multimorbid, chronically ill patients with MCI during the COVID-19 pandemic, this result is highly valuable. Furthermore, our results on relevant acceptance and usability factors of such a telemonitoring app provide important information for the design and implementation of future home-based telemedicine solutions. Our study design and results can be used as a starting point for quantitative studies in this field with a larger sample size and a controlled study design.

In addition to its strengths, our study also has limitations. The recruitment of patients from this vulnerable group was difficult because of concerns regarding their own abilities to operate a telemonitoring app; hence, the occurrence of a selection bias cannot be excluded. Moreover, some patients from this cohort dropped out of the overall study before the qualitative study started due to technical difficulties with the telemonitoring app examined. Finally, we included all 12 patients who met the inclusion criteria and agreed to be interviewed to achieve the greatest heterogeneity possible within our study sample. During the interview series, we noticed that the patients’ responses were repeated toward the end and we reached saturation concerning the topics addressed in the interviews with our available sample. However, it cannot be excluded that the inclusion of further patients with MCI would have opened other relevant topics. When interviewing study participants, there is always the possibility that their answers are influenced by social desirability, which in turn could lead to biased results. To tackle this issue, we opted for an open interview setting, gave the participants the chance to ask questions, and kept the number of people present during the interview to a minimum. In addition, recall bias can exist, particularly in the case of interviewing patients with MCI. Some patients did not adhere to the interview topics and talked about aspects subjectively perceived to be relevant. Hence, some questions remained unanswered or may have been answered inadequately (response bias). However, our study design and results provide a valuable basis for future studies with a larger sample size.

### Conclusions

When continuous personal support was available right from the start of use and when the app was tailored in a needs- and disease-specific design, ensuring high perceived ease of use, the majority of examined patients with MCI were capable of operating the telemonitoring app independently. Hence, this study provides initial evidence that older patients with multimorbidity and MCI could increasingly be considered as a relevant user group for telemonitoring apps and should be involved as codesigners in their development. Future studies should investigate this issue further with a larger sample of patients with MCI.

## Appendix

Multimedia Appendix 1 Interview guideline.

## Abbreviations

ATMoSPHAERE: Autonomy despite multimorbidity in Saxony through patient empowerment, holistic care for older people with networking of all regional institutions and service providers
MCI: mild cognitive impairment
MMSE: Mini-Mental State Examination
OECD: Organisation for Economic Co-operation and Development
