# Supplementary material for: Independent Use of a Home-Based Telemonitoring App by Older Patients With Multimorbidity and Mild Cognitive Impairment: Qualitative Study
Source: JMIR Hum Factors. 2021 Jul 12;8(3):e27156. doi: 10.2196/27156 (PMC8314150; doi:10.2196/27156)
Supplement: Multimedia Appendix 1 [file humanfactors_v8i3e27156_app1.pdf]

Interview Guideline

**Icebreaker question and first phase of using the telemonitoring app**
**You have been using the telemonitoring app for two months now.**<br>1. Think back to when you first started using it: What was your first impression of the app?<br>2. How satisfied are you with the use of the app so far?<br>3. Did you find it rather easy or rather difficult to understand how to use the app?<br>What exactly did you find easy? What was particularly easy for you to understand?<br>What exactly was difficult for you? What was particularly difficult for you to understand?<br>4. Did you feel rather safe or rather insecure when using the app?<br>Insecure - What unsettled you? What do you think needs to be changed to reduce this uncertainty?<br>When the system was installed at your home and you received the tablet, a German Red Cross employee explained how to use it.<br>5. In addition to the initial introduction by the German Red Cross employee, did you have support in learning how to use it?<br>YES - Who did you contact in this regard? Who supported you (family, friends, German Red Cross staff)?
**Ease of use of the telemonitoring app**
6. Do you currently find it rather easy or rather difficult to use the app?<br>DIFFICULT - What exactly do you find difficult or complicated to understand?<br>EASY - What exactly do you find easy or simple to understand?
**Comprehensibility of outputs/displays/terms/symbols of the app**
7. Are the outputs and displays you see when using the app understandable to you so that you can understand them without further support or explanation, or is this not the case?<br>NO - What exactly is difficult for you to understand?<br>What do you think the reason for this is?<br>8. Are the terms and symbols you see when using the app understandable to you or rather less so?<br>NO - Can you tell me (or show me) which terms and symbols are specifically not understandable to you?<br>What do you think the reason for this is?

Assessment of contents/functionalities of the telemonitoring app

9. How do you rate the range of functions the app offers?

   What features are you missing that would be useful to you? Could you please explain this in more detail: Why would these functions be useful to you?

   Which functions offered by the app do you consider to be superfluous/unnecessary? Could you please explain this in more detail: Why do you consider these functions to be superfluous/unnecessary?

10. Do you feel that the app is too complicated for the range of functions it offers or do you find that this ratio is balanced?

Assessment of the content presentation of the telemonitoring app

Now we will take a closer look at how the content you see in the app is displayed:

11. How do you rate the size of the images?
12. How do you rate the font size?
13. How do you rate the color contrast?

User-system interaction / guiding the user through the app

14. How would you generally rate the interaction with the app?
15. Is it rather easy or rather difficult for you to navigate to the important points in the app?

    DIFFICULT - Can you tell me what exactly makes it difficult for you / what is complicated for you?

16. How do you feel about the effort required to make entries? Do you find this to be little or is it very time-consuming?

    MUCH EFFORT - Why do you think it is so time-consuming to make entries?

    NOT MUCH EFFORT - Why do you think it is so easy to make entries?

17. Is the information/feedback from the app understandable or is it difficult to understand?

    DIFFICULT - Can you remember which information/feedback from the app you found particularly difficult to understand and why?

18. How did you cope with the app's reaction to any accidentally entered wrong values?

Individual perceived additional benefit / influence on therapy management

19. Do you feel that the use of the app has an influence on how you cope with your diseases in everyday life?

    YES - Could you please describe this influence in more detail?

      What has improved by using the app?

      Have things developed negatively by using the app? If so, could you please explain this in more detail?

NO - What do you think could be the reason for this?<br>20. Has the use of the app made your therapy management / daily routine easier or more difficult?<br>YES - What exactly has been simplified?<br>NO - What exactly has become more difficult/complicated?
21. What other technologies (e.g. smartphone, tablet, PC/laptop) apart from the telemonitoring app do you use regularly?
